# The characteristics of the spectra of superior venae cavae in patients with right heart failure

**DOI:** 10.1186/1476-7120-4-21

**Published:** 2006-04-07

**Authors:** Hua-Ping Jia, Yun-You Duan, Tie-Sheng Cao, Li-Jun Yuan, Juan Li

**Affiliations:** 1 Tangdu Hospital, The Fourth Military Medical University, Xi an, Shaanxi province, China

## Abstract

**Background:**

Aimed to elucidate the characteristics of the spectra of superior venae cavae (SVC) in respiratory cycles in patients with right heart failure.

**Methods:**

The spectra of SVC of 30 patients with right heart failure and 30 paired healthy subjects were recorded through right supraclavicular fossa view. The profiles of spectra of superior venae cavae were observed, and peak velocity and velocity time integral (VTI) of every wave of SVC under spontaneous respiration were measured for statistical analysis.

**Results:**

In healthy subjects, the peak velocities and VTI of S wave and D wave increased in inspiratory phase and diminished in expiratory phase, and which of S wave were larger than which of D wave in whole respiratory cycle. In patients with right heart failure, spectral variations of SVC could be classified into three patterns: Pattern I: peak velocities and VTI of S wave were larger than that of D wave in early inspiratory phase, but peak velocities and VTI of D wave were larger than those of S wave in late inspiratory phase and early expiratory phase [Pattern I-1], even in whole respiratory cycle [Pattern I-2]; Pattern II: the S wave disappeared and was substituted by inverse wave with low amplitude in whole respiratory cycle. Pattern III: the profiles of the spectra of SVC in patients were similar to those of healthy subjects. In the whole, the respiratory variation ratios of peak velocities and VTI of S wave and D wave were diminished in patients compared with those in healthy subjects.

**Conclusion:**

The spectra of superior venae cavae in patients with right heart failure were abnormal, and these characteristics could be used as signs in evaluating right heart failure.

## Background

The profiles of spectra of superior venae cavae of healthy subjects have been described, and the factors which affected them have been recognized, such as age, sex, respiration, and so on [1,2]. Among all these factors, respiration was the most important one. Some scholars have described the respiratory variations about the spectra of superior venae cavae of healthy subjects, and the respiratory variations of the spectra of superior venae cavae in some pathological conditions have been studying, but few are about the respiratory variations of the spectra of superior venae cavae in patients with right heart failure.

## Methods

### Clinical data

30 consecutive patients with right heart failure and their paired healthy subjects were enrolled in this study. Because the functional grading standard of right heart has not been established as yet, NYHA cardiac functional grading standard was adopted instead. The patients were selected according to these standards as follows: 1. NYHA cardiac functional grading standard III-IV; 2. accompanying definite congestion of systemic circulation, which embraced distension of jugular veins and hepatic veins, edema of lower extremity and dilatation of right heart. Patients would be excluded if they had the diseases as follows: 1. severe arrhythmia; 2. moderate-above pericardial effusion; 3. chronic pulmonary diseases; 4. obstructive diseases of superior vena cava. According to these standards above, 18 male and 12 female patients were enrolled, including 12 patients with dilated cardiomyopathy, 8 patients with ischemic cardiomyopathy and 10 patients with valve diseases. The average age was 48 years old. Thirty healthy subjects in control group were paired with patients according to age and sex.

### Examination methods

Applying sequoia-512 diasonography (transducer 3V2c or 7V3c, 3.5 MHz), the spectra of superior venae cavae of 30 patients and 30 paired healthy subjects were recorded through right supraclavicular fossa. Electrocardiogram and respiratory curve were recorded simultaneously. S wave and D wave were defined as the major waves, and the spectra of superior vena cava with maximum and minimum peak velocities of the major waves in respiratory cycle were selected for analysis. Peak velocities and velocity time integral (VTI) of every wave of superior venae cavae were measured, and their respiratory variation ratios were calculated. The respiratory variation ratios of peak velocities and VTI embraced inspiratory variation ratio and expiratory variation ratio. Inspiratory variation ratio was defined as the peak velocities (or VTI) in inspiratory phase subtracted it in expiratory phase, and then divided by it in expiratory phase, and expiratory variation ratio was defined as the peak velocities (or VTI) in inspiratory phase subtracted it in expiratory phase, and then divided by it in inspiratory phase.

### Statistical analysis

Data were processed by paired-samples T test with Statistical Package for Social Sciences (SPSS) 11.0, and p < 0.05 was considered statistically significant.

## Results

**Figure 1:**
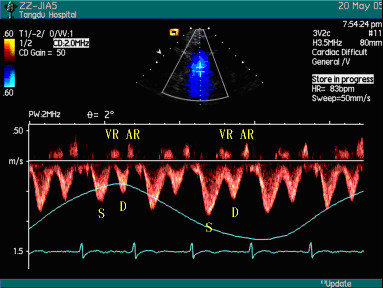
The spectra of SVC of healthy subjects in the respiratory cycle [Pattern III].

The spectra of superior venae cavae of healthy subjects consisted of four waves typically, which were S wave and VR wave in systolic phase, D wave in diastolic phase, and AR wave in atrial systolic phase. The spectra of superior venae cavae of healthy subjects varied regularly in respiratory cycle. Peak velocities and velocity time integrals (VTI) of S wave and D wave increased in inspiratory phase and diminished in expiratory phase, and the VR wave and AR wave varied inversely. Peak velocities and VTI of S wave were larger than those of D wave in whole respiratory cycle in healthy subjects (Figure 1).

**Figure 2:**
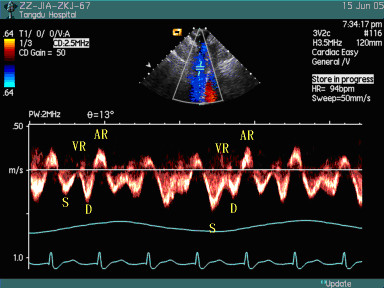
The spectra of SVC in the respiratory cycle in patients with right heart failure [Pattern I-1].

**Figure 3:**
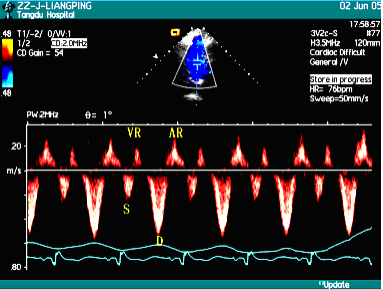
The spectra of SVC in the respiratory cycle in patients with right heart failure [Pattern I-2].

**Figure 4:**
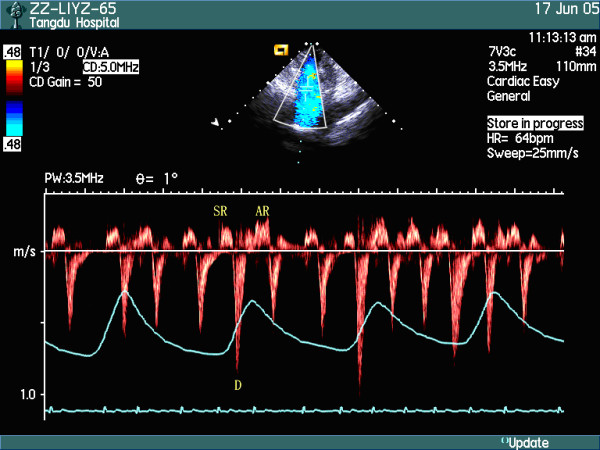
The spectra of SVC in the respiratory cycle in patients with right heart failure [Pattern II].

The spectra of superior venae cavae in patients with right heart failure could be classified into three patterns: Pattern I: peak velocities and VTI of S wave were larger than those of D wave in early inspiratory phase, but peak velocities and VTI of D wave were larger than those of wave S in late inspiratory phase and early expiratory phase (Figure 2), even in whole respiratory cycle (Figure 3). Twelve patients belonged to this pattern; Pattern II: the systolic anterior S wave disappeared and was substituted by inverse wave with low amplitude (Figure 4), and 8 patients belonged to this pattern. Pattern III: the profiles of the spectra of SVC in patients were similar to those of health subjects, and 10 patients belonged to this pattern. Peak velocities and VTI of every wave and their respiratory variation ratios in two groups were listed in Table 1 to Table 4, and the results of statistical analysis were as follows: there were no differences in the peak velocities of S wave in respiratory cycle and its expiratory variation ratios between the patient group and the control group (*P *> 0.05), but the inspiratory variation ratios of S wave were larger in the control group than in the patient group (*P *< 0.05); there were no differences in VTI of S wave in respiratory cycle and its inspiratory variation ratios between the patient group and the control group (*P *> 0.05), but the expiratory variation ratios of VTI of S wave were larger in the control group than in the patient group (*P *< 0.05). There were no differences in the peak velocities of D wave in inspiratory phase between the patient group and the control group (*P *> 0.05), but the peak velocities of D wave in expiratory phase were larger in the patient group than in the control group (*P *< 0.05), and the respiratory variation ratios of peak velocities of D wave were larger in the control group than in the patient group (*P *< 0.05); there were no differences in the VTI of D wave in respiratory cycle and its expiratory variation ratios between the patient group and the control group (*P *> 0.05), but the inspiratory variation ratios of VTI of D wave were larger in the control group than in the patient group (*P *< 0.05). The peak velocities and VTI of VR wave were larger in the patient group than in the control group in whole respiratory cycle (*P *< 0.05), but there were no differences in their respiratory variation ratios. The peak velocities and VTI of AR wave were larger in the patient group than in the control group in expiratory phase (*P *< 0.05), but there were no differences in inspiratory cycle; the respiratory variation ratios of peak velocities and the expiratory variation ratios of VTI of AR wave were larger in the patient group than in the control group (*P *< 0.05), but there were no differences in the inspiratory variation ratios of VTI of AR wave between the two groups (*P *> 0.05).

**Table 1:** Peak velocities and VTI of S wave and their respiratory variation ratios ( ± *s*).

	Patients		Health subjects	
		
	Peak velocity (cm/s)	VTI(mm)	Peak velocity (cm/s)	VTI(mm)
Inspiration	53.00 ± 20.19	0.1038 ± 0.0622	63.30 ± 17.07	0.1647 ± 0.0513
Expiration	39.63 ± 13.48	0.0724 ± 0.0359	43.80 ± 13.88	0.1028 ± 0.0291
Inspiratory variation ratio (%)	32.26 ± 16.49	39.06 ± 34.04	49.67 ± 36.30	68.79 ± 39.64
Expiratory variation ratio (%)	23.32 ± 9.13	24.80 ± 14.57	29.98 ± 14.02	34.71 ± 17.12

There were no differences in the peak velocities of S wave in respiratory cycle and its expiratory variation ratios between the patient group and the control group (*P *> 0.05), but the inspiratory variation ratios of S wave were larger in the control group than in the patient group(*P *< 0.05); there were no differences in VTI of S wave in respiratory cycle and its inspiratory variation ratios between the patient group and the control group (*P *> 0.05), but the expiratory variation ratios of VTI of S wave were larger in the control group than in the patient group(*P *< 0.05).

## Discussion

The normal pattern of spectrum of superior vena cava and its respiratory variation have been accepted by most scholars, which were characterized of predominant systolic wave, namely peak velocity of S wave were larger than that of D wave in whole respiration cycle, and it was consistent with our study [3]. Up to now, the variations of spectrum of superior vena cava under most pathological conditions have not been elucidated, and recent studies highlighted the prospect of examination of spectrum of superior vena cava. The spectrum of superior vena cava could be affected by many physiological factors, and respiration was the most apparent one. Recent studies proved the significance of respiratory variation in the examination of superior vena cava. Izumi and his colleagues [4-6] made serial studies on the spectra of superior venae cavae in chronic pulmonary diseases, and categorized the spectra of superior venae cavae of the patients with chronic pulmonary diseases into two patterns: one took on the normal mode like that of the health subjects, and the other with disappearance of D wave or both S wave and D wave in the expiratory phase.

The pattern of venous return in patients with pulmonary disease varied depending on the mode of the ventilation disturbance and the presence of right ventricular pressure overload. The abnormal patterns were observed in some of the patients with obstructive ventilation disturbance and most of the patients with combined ventilation disturbance, and the right atrial pressure exceeded the subclavian vein pressure in the expiratory phase in cases with abnormal patterns. One study of Byrd [7] indicated respiratory variation in flow velocities of superior vena cava increased in patients with hemodynamically insignificant pericardial effusions and greatest in patients with cardiac tamponade. In obstructive diseases of superior vena cava, respiratory variation of the spectra of superior venae cavae diminished or even vanished, which was a significant sign in diagnosing these diseases [8]. Because the spectra of superior vena cava were affected in these diseases above, these patients were excluded in our study.

In this study, abnormal patterns of spectra of superior venae cavae were observed in patients with right heart failure, which had some differences from those of the diseases above. One study on the patients with congestive heart failure found the similar abnormal spectral pattern of superior vena cava, and indicated the pattern with predominant diastolic wave identified patients with a reduced right ventricular ejection fraction and elevated right atrial pressure (>8 mmHg), so the pattern of superior vena cava was a useful tool to estimate the extent of the right circulatory impairment in patients with congestive heart failure [9].

Though the results of our study indicated that the spectral patterns of superior vena cava had their own characteristics in some patients with right heart failure, others had similar spectral pattern to that of health people, in this case, the difference of respiratory variations between two groups could be used as additional features. Respiratory variations of every wave and its VTI were complicated. The peak velocities and VTI of S wave and D wave overlapped in two groups, but their respiratory variation ratios mostly diminished significantly in patient group, and it could be the evidence of right heart failure that had not been reported before. Although the respiratory variation ratios of S wave and D wave could not be the individual index, they should be an important additional index to evaluate right heart failure. There were some differences in respiratory variations of VR wave and AR wave between two groups, particularly in AR wave, and their respiratory variations were contrary to those of S wave and D wave. These two waves were of low amplitude, and their respiratory variations were not apparent as those of S wave and D wave, their clinical values needed further study.

At present, there was no functional grading standard of right heart available, and NYHA cardiac functional grading standard was adopted instead in this study. In order to confirm right heart failure, some additional conditions were added and they might bring up some deflections. On the other hand, right heart failure often accompanied with pulmonary artery hypertension, tricuspid insufficiency, and elevated right heart pressure, and these factors were often not parallel in one patient and could affect the spectrum of superior vena cava respectively, so there were complicated factors which could affect the results. Even so, this study indicated that the spectra of superior venae cavae in patients with right heart failure had their own characteristics, and these characteristics could be used to evaluate right heart failure. Further studies needed to be done in detail.

## Conclusion

In patients with right heart failure, the spectra of superior vena cava were abnormal, and it could be a new view to evaluate right heart function through observing the spectrum of superior vena cava.

## Competing interests

The author(s) declare that they have no competing interests.

## Authors' contributions

JIA carried out the clinical investigation, performed the statistical analysis, and drafted the manuscript. DUAN designed the experiment and proofread the paper. CAO participated in the design of the study. YUAN and LI participated in the clinical investigation, and helped to draft the manuscript. All authors read and approved the final manuscript.

**Table 2:** Peak velocities and VTI of D wave and their respiratory variation ratios ( ± *s*).

	Patients		Health subjects	
		
	Peak velocity (cm/s)	VTI(mm)	Peak velocity (cm/s)	VTI(mm)
Inspiration	39.67 ± 26.52	0.0684 ± 0.0509	38.00 ± 9.15	0.0747 ± 0.0371
Expiration	34.43 ± 19.21	0.0580 ± 0.0354	24.73 ± 9.12	0.0454 ± 0.0243
Inspiratory variation ratio (%)	27.03 ± 16.34	33.72 ± 30.33	65.34 ± 44.38	86.18 ± 66.74
Expiratory variation ratio (%)	20.39 ± 13.40	29.98 ± 24.82	35.00 ± 17.96	45.16 ± 35.49

There were no differences in the peak velocities of D wave in inspiratory phase between the patient group and the control group (*P *> 0.05), but the peak velocities of D wave in expiratory phase were larger in the patient group than in the control group (*P *< 0.05), and the respiratory variation ratios of peak velocities of D wave were larger in the control group than in the patient group (*P *< 0.05); there were no differences in the VTI of D wave in respiratory cycle and its expiratory variation ratios between the patient group and the control group (*P *> 0.05), but the inspiratory variation ratios of VTI of D wave were larger in the control group than in the patient group (*P *< 0.05).

**Table 3:** Peak velocities and VTI of VR wave and their respiratory variation ratios ( ± *s*).

	Patients		Health subjects	
		
	Peak velocity (cm/s)	VTI(mm)	Peak velocity (cm/s)	VTI(mm)
Inspiration	17.15 ± 6.27	0.0159 ± 0.0110	12.77 ± 3.34	0.0080 ± 0.0043
Expiration	19.74 ± 6.71	0.0192 ± 0.0157	15.13 ± 3.90	0.0098 ± 0.0049
Inspiratory variation ratio (%)	16.47 ± 5.51	21.55 ± 12.79	15.75 ± 8.90	17.69 ± 17.08
Expiratory variation ratio (%)	20.21 ± 7.94	27.68 ± 18.19	20.10 ± 14.45	28.25 ± 34.71

The peak velocities and VTI of VR wave were larger in the patient group than in the control group in whole respiratory cycle (*P *< 0.05), but there were no differences in their respiratory variation ratios.

**Table 4:** Peak velocities and VTI of AR wave and their respiratory variation ratios ( ± *s*).

	Patients		Health subjects	
		
	Peak velocity (cm/s)	VTI(mm)	Peak velocity (cm/s)	VTI(mm)
Inspiration	18.33 ± 5.29	0.0173 ± 0.0103	17.40 ± 5.82	0.0134 ± 0.0069
Expiration	23.08 ± 7.78	0.0228 ± 0.0119	19.23 ± 5.98	0.0154 ± 0.0075
Inspiratory variation ratio (%)	21.71 ± 16.02	29.79 ± 22.95	9.83 ± 8.81	27.83 ± 40.52
Expiratory variation ratio (%)	29.65 ± 24.91	47.21 ± 46.41	12.11 ± 12.92	34.71 ± 17.12

The peak values and VTI of AR wave were larger in the patient group than in the control group in expiratory phase (*P *< 0.05), but there were no differences in inspiratory cycle; the respiratory variation ratios of peak values and the expiratory variation ratios of VTI of AR wave were larger in the patient group than in the control group (*P *< 0.05), but there were no differences in the inspiratory variation ratios of VTI of AR wave between the two groups (*P *> 0.05).
